# Metabolic dysfunction associated fatty liver disease and type 2 diabetes: pathophysiological links, epidemiological trends, and clinical implications

**DOI:** 10.3389/fendo.2025.1669478

**Published:** 2025-10-27

**Authors:** Mohammad Sarif Mohiuddin, Noushin Tabassum Neha, Jawad Ul Karim Mahir, Fardin Al Fahad Shakib, Md. Ashraful Alam, Md. Wahiduzzaman, Rashu Barua, Shakila Jahan Shimu, Mahbubur Rahman, Md. Rafin Hossain, Mohammad Hossain Shariare, Mohammad Mohabbulla Mohib, Mohammad Borhan Uddin

**Affiliations:** ^1^ Department of Foundations of Medicine, New York University Grossman Long Island School of Medicine, Mineola, NY, United States; ^2^ Department of Pharmaceutical Sciences, North South University, Dhaka, Bangladesh; ^3^ Department of Health Informatics, Harrisburg University of Science and Technology, Harrisburg, PA, United States; ^4^ Julius Bernstein Institute of Physiology, Medical School, Martin Luther University of Halle-Wittenberg, Halle, Germany

**Keywords:** metabolic dysfunction-associated fatty liver disease (MASLD), non-alcoholic fatty liver disease (NAFLD), type 2 diabetes mellitus (T2DM), insulin resistance (IR), hepatic steatosis, lipotoxicity, inflammation, adipokines

## Abstract

Metabolic dysfunction-associated steatotic liver disease (MASLD), previously known as non-alcoholic fatty liver disease (NAFLD), has recently been recognized as a public health issue because it is closely linked to metabolic disorders, including type 2 diabetes mellitus (T2DM). This review aims to discuss the bidirectional relationship between MASLD and T2DM and the similarities in their pathophysiology, which include insulin resistance (IR), lipogenesis, inflammation, and alterations in the gut microbiota. The incidence of MASLD has increased concomitantly with the obesity and diabetes pandemic, and more than 60% of individuals with T2DM have liver steatosis. The metabolic dysfunction is followed by adipokines, inflammatory mediators like TNF-α, IL-6, and oxidative stress, which worsen NAFLD and lead to T2DM. Since MASLD is usually asymptomatic in its early stages, it is important to screen high-risk populations such as obese and metabolic syndrome patients to enable them to start treatment early. Lifestyle changes, including changes in diet, weight loss, and increased physical activity, are currently the mainstay of treatment for MASLD; however, the potential of new pharmacological approaches that act on insulin signaling, hepatic lipid metabolism, and inflammation to improve treatment is encouraging. Although the role of MASLD in the pathogenesis of T2DM has been well-documented, there are issues with standardizing the diagnostic criteria and the availability of effective treatments. This is because the multidisciplinary management of metabolic diseases needs hepatology, endocrinology, and public health measures to prevent a global epidemic. More studies are required to fully understand the underlying molecular mechanisms of MASLD-T2DM and search for specific treatment for high-risk patients.

## 1 Introduction

Metabolic dysfunction-associated steatotic liver disease (MASLD) is currently one of the most important public health issues in the world because it is directly related to metabolic disorders such as obesity, insulin resistance, and type 2 diabetes mellitus (T2DM) (1, 2). MASLD was proposed as a new name for Nonalcoholic fatty liver disease (NAFLD) in order to better reflect the metabolic basis of the disease and to emphasize the relationship between the liver and the rest of the body (3). NAFLD is different from MASLD in that it does not need the exclusion of other causes of liver disease, including alcohol and viral hepatitis (4). It is diagnosed by the presence of hepatic steatosis in obese, T2DM, or two or more metabolic risk factors: increased waist circumference, raised triglycerides, low HDL-cholesterol, hypertension, or prediabetes (2, 5, 6). Nevertheless, although the use of the MASLD definition is increasing, so is the controversy among hepatologists and diabetologists regarding its definition, especially regarding its staging and management (7).

MASLD is particularly notorious for being able to accelerate the development of T2DM, a chronic metabolic disease characterized by hyperglycemia, IR, and beta cell failure (8). Epidemiological data shows that MASLD and T2DM are mutually exclusive conditions that worsen the features of the other and lead to worse outcomes (9–12). Obesity is a classical risk factor for both MASLD and T2DM (9), but increasing data indicate that MASLD can also develop among lean people, also called ‘lean NAFLD’ (13). These subjects may not have metabolic syndrome but will have hepatic steatosis, inflammation and enhanced sensitivity to develop T2DM (14–16).

The main pathophysiological mechanisms through which Insulin resistance (IR), hepatic lipid accumulation, and low-grade inflammation lead to MASLD and T2DM have been described (17, 18). MASLD is characterized by hepatocellular lipid overloading, which causes IR and leads to insulin secretion, which in turn increases hepatic lipid content and systemic metabolic disturbances. This negative feedback increases the risk of developing T2DM (19). Moreover, hepatic fibrosis, which is a well-known complication of progressive MASLD, is an independent predictor of incident T2DM, thus suggesting that long-term liver dysfunction is an important cause of diabetes (17, 18, 20). Furthermore, Hepatocytes plays the central role in glucose homeostasis, lipid metabolism, and inflammatory signaling and, therefore, has a crucial role in the overall metabolic status of the organism (21–23). Hepatic steatosis, when present for a prolonged duration, leads to changes in lipid metabolism, increased hepatic glucose production, and blunted insulin signaling, ultimately contributing to metabolic deterioration (23). Furthermore, the gut-liver axis, which is activated by dysbiosis and increased intestinal permeability, has been shown to participate in the pathogenesis of MASLD and T2DM, linking hepatic inflammation to metabolic disorders (23). These multifactorial interactions underscore the importance of considering MASLD as a precipitating factor and active participant in the pathophysiology of T2DM. In addition, studies show that complications of MASLD are not limited to hepatobiliary sequelae but also include CVD, chronic kidney disease, and an enhanced tendency to cancer (23–25). Patients with MASLD and T2DM are known to have a higher mortality rate due to hepatic and extrahepatic complications (25). Since most cases of early MASLD are asymptomatic, many people remain undiagnosed until the metabolic impairment is severe, which highlights the importance of screening and intervention (26). Despite the available evidence highlighting the relationship between MASLD and T2DM, many unanswered questions remain regarding the specific pathways through which this relationship is established and the potential treatment objectives. The absence of standardized guidelines for managing MASLD adds complexity to the management of the condition and its progression to T2DM. Although lifestyle changes, including alteration in diet and increase in physical activity, are recommended for the management of MASLD (27), pharmacological therapy aimed at hepatic lipid metabolism, inflammation, and fibrosis is currently under consideration as a potential treatment option. Thus, more studies are needed to fully understand the consequences of untreated MASLD on diabetes development and to find ways to prevent or lessen the impact of both diseases. These challenges shall be of great importance in the management of patients with metabolic diseases with the aim of enhancing the quality of life and preventing the continued rise of metabolic diseases globally. This may provide opportunities for early identification of people with high risk of developing T2DM and thus help in prevention of the increasing incidence of metabolic diseases.

## 2 Epidemiological data

With the prevention and control of many infectious diseases, Non-Communicable Diseases (NCDs) have become the leading global diseases burden (28, 29). Metabolic syndrome and its comorbidities, including T2DM and MASLD are now of major interest (17, 18). The prevalence of T2DM has risen sharply; the International Diabetes Federation (IDF) estimated that 527 million adults (10.5%) aged 20–79 years had diabetes in 2021 and is expected to rise to 643 million in 2030 and 783 million in 2045 (30). T2DM is the most common type of diabetes, affecting more than 90% of diabetes cases and straining healthcare systems. By 2045 it is predicted that the cost of diabetes will be greater than $1,054 billion, highlighting the need for preventive measures (30–32). Also, diabetes is a major cause of ischemic heart disease and stroke which are the leading causes of death globally (33).

### 2.1 Global prevalence and trends of MASLD and T2DM

MASLD has recently become the leading cause of chronic liver disease (CLD) and is now recognized as the most common liver disease (26, 34). It has been estimated that MASLD affects 32% of the global adult population and the prevalence is increasing (35). MASLD complications such as NASH, cirrhosis and HCC are the leading causes of liver related morbidity and mortality (36). Moreover, MASLD has been observed in more than 38% of the global population, with 5.37% having a lean phenotype and 29.78% having a non-obese phenotype (37). As MASLD is highly associated with metabolic disorders, it is regarded as one of the major causes of T2DM.

Epidemiological studies indicate that MASLD is increasing rapidly worldwide, with some studies reporting a prevalence of over 40% in high-risk male populations, compared to approximately 26% in females (38). The incidence of MASLD has been on the rise concurrently with the obesity pandemic, and it is still rising in countries with an increased sedentary lifestyle and poor dietary choices. The increase in the consumption of processed foods containing high levels of refined carbohydrates and saturated fats has also contributed to the increasing incidence of metabolic diseases, including both MASLD and T2DM (39).

It has been found that MASLD boosts the risk of developing T2DM by two to five times according to the level of hepatic fibrosis and metabolic dysfunction (40). Liver fibrosis is a marker of poor prognosis and it is known that patients with MASLD are at higher risk of developing diabetes. For instance, a large prospective cohort study of 365,339 MASLD patients with no history of T2DM at baseline found that 8,774 developed T2DM during an 11-year follow-up, thus supporting the notion that MASLD occurs before the onset of diabetes (41).

### 2.2 Ethnic and racial disparities in MASLD and T2DM prevalence

Racial and ethnic inequalities in the rates of MASLD and T2DM have been noted. The Hispanic population has the highest prevalence of MASLD (37.0%) in the United States, and the non-Hispanic Black population has a lower prevalence (24.7%) than the non-Hispanic White population (29.3%) (42). In Asia, the rate of MASLD is also the highest among the Uyghur population in China (46.6%) than Han Chinese (29.3%), Kazakhs (24.3%) and Mongolians (25%) (43). Turkish adults have the highest prevalence of MASLD among all European countries at 48.4% (44). The prevalence of MASLD and NASH is also rising; nonetheless, the disease burden varies by region, with the highest burden seen in Latin America (44.37%), South Asia (33.83%), and the Middle East (36.53%) (42).

A meta-analysis and systematic review revealed that the prevalence of MASLD is markedly higher in high-risk populations, including obese, metabolically active, or insulin-resistant individuals, than in the general population (45). The frequency may be more than 50% in such persons, especially in countries with a high prevalence of obesity, including the United States and the Middle East (46).

The contribution of genetic and environmental determinants to the disease has been identified as an important factor. Some genetic variants, for example, the PNPLA3 polymorphism, have been linked to an increased risk of MASLD and T2DM in Hispanic and Asian peoples (42). This genetic predisposition, together with lifestyle, may help to explain the different prevalence rates observed across different ethnic groups.

### 2.3 The bidirectional relationship between MASLD and T2DM

The close association between MASLD and T2DM has been widely studied, with epidemiological data demonstrating a bidirectional relationship (41). A meta-analysis by Mantovani et al. involving over 500 studies found that patients with MASLD, diagnosed via ultrasonography, had more than twice the risk of developing T2DM compared to those without MASLD (47). The severity of hepatic fibrosis further increased this risk. Elevated liver enzymes such as alanine aminotransferase (ALT), aspartate aminotransferase (AST), and gamma-glutamyl transferase (GGT) have also been identified as significant predictors of T2DM risk, independent of BMI (48, 49).

Conversely, some studies suggest that T2DM contributes to MASLD’s development and progression. Younossi et al. reported that over 55% of adult T2DM patients have MASLD, with 37.3% developing NASH and 17% progressing to advanced fibrosis (50). Geographical differences further influence this relationship, with European diabetics exhibiting the highest MASLD prevalence (68%) (1). An observational study employing transient elastography found that over 50% of T2DM patients had severe hepatic steatosis, while 20% exhibited advanced fibrosis. Genetic studies have also suggested a causative link, with genome-wide association studies indicating that genetic predisposition to T2DM mediates approximately 51.4% of BMI’s effect on MASLD risk (51).

Epidemiological disparities in MASLD prevalence reflect underlying molecular susceptibilities. For instance, the disproportionately high prevalence of MASLD among Hispanic populations corresponds to a higher frequency of the PNPLA3 I148M variant, which promotes triglyceride retention in hepatocytes and aggravates insulin resistance (52). Similarly, the rapid rise in MASLD in South Asia is linked to dietary shifts toward refined carbohydrates and saturated fats, which drive mTORC1–SREBP1c activation and lipotoxicity (53, 54). Lean MASLD, increasingly observed in Asian populations, illustrates how genetic predisposition and subtle adipose tissue dysfunction can precipitate hepatic steatosis and subsequent β-cell stress, even in the absence of obesity (55–58). Thus, epidemiological trends are not isolated observations but manifestations of molecular pathways, including genetic polymorphisms, inflammatory signaling, and altered lipid handling, which predispose populations to differential risks of MASLD and T2DM.

### 2.4 Pathophysiology linking MASLD to T2DM development

**Figure 1:**
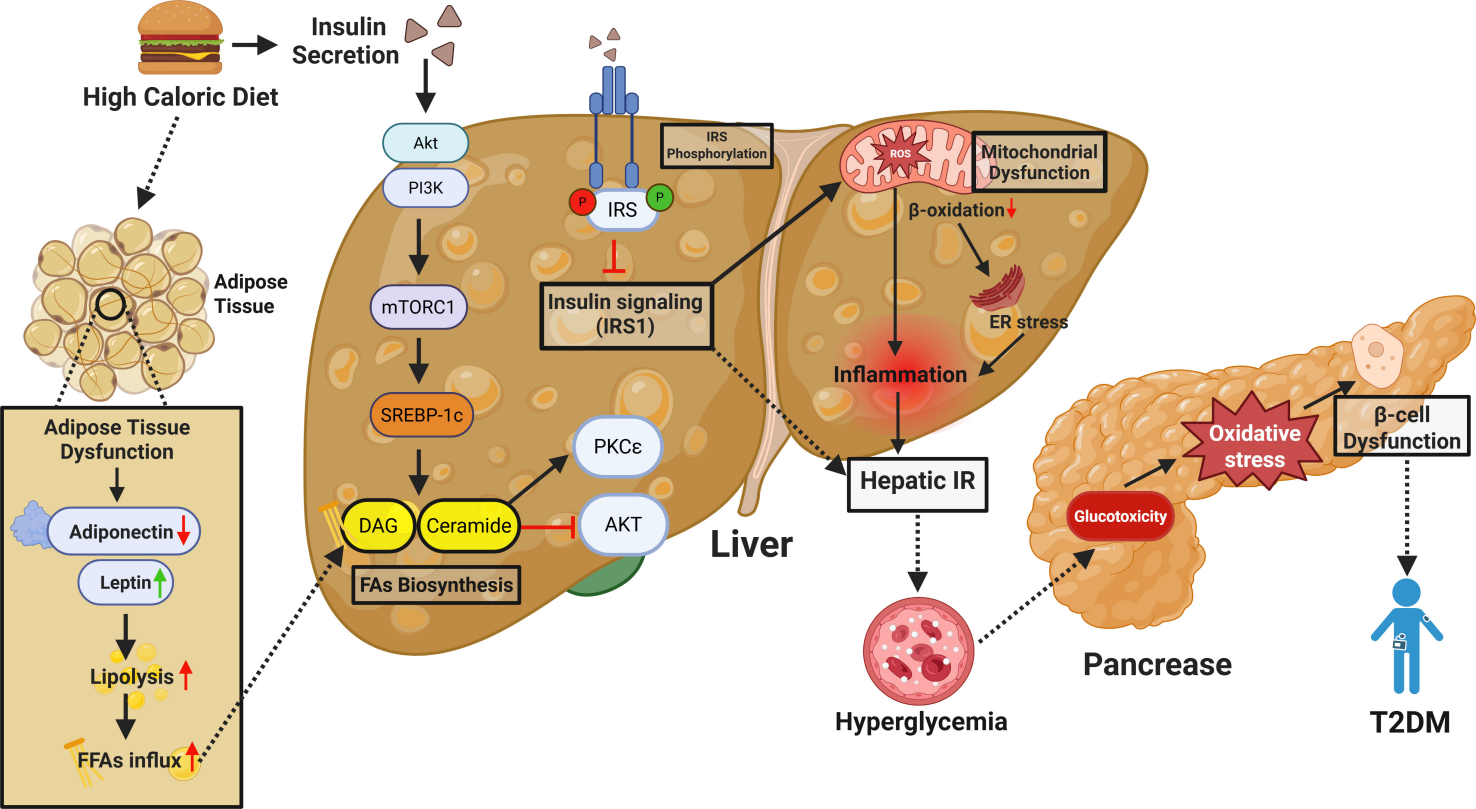
Mechanistic overview of high-caloric diet-induced hepatic insulin resistance and its contribution to T2DM. This schematic illustrates how chronic high-calorie intake drives hepatic insulin resistance (IR) and contributes to T2DM development. Excess nutrients disrupt adipose tissue function—reducing adiponectin, increasing leptin, and elevating free fatty acids (FFAs). These FFAs accumulate in the liver and are converted into lipotoxic intermediates like diacylglycerol (DAG) and ceramide, which activate PKCϵ and impair insulin signaling by inhibiting IRS1 phosphorylation. Overactivation of mTORC1 and SREBP-1c further promotes lipogenesis and lipid buildup. Mitochondrial dysfunction and impaired β-oxidation increase ROS and ER stress, driving hepatic inflammation and IR. This leads to reduced glucose uptake and increased hepatic glucose production, causing systemic hyperglycemia. Chronic hyperglycemia induces glucotoxicity and oxidative stress in pancreatic β-cells, impairing insulin secretion. Together, hepatic IR and β-cell dysfunction drive T2DM progression.

The link between MASLD and T2DM extends beyond mere coexistence, as both conditions share common pathophysiological mechanisms, primarily insulin resistance and compensatory hyperinsulinemia, which lead to metabolic dysfunction and β-cell failure (7, 17, 47, 51). For instance, a meta-analysis of 117,020 MASLD patients over 5 years of follow-up reported a nearly two-fold increased risk of T2DM occurrence (59). Lifestyle factors such as sleep have also been implicated in diabetes risk among MASLD patients, and lack of sleep, insomnia, snoring, and daytime sleepiness are all associated with increased risk of T2DM. The evolution of MASLD to T2DM is mainly through hepatic insulin resistance that affects glucose metabolism and systems-level metabolic dysfunction (1, 17, 18, 20, 26, 59). Insulin resistance in the liver, lipotoxicity, and chronic inflammation lead to β cell stress and failure, which is characteristic of T2DM. In addition, the manifestation of adipose tissue dysfunction, with increased production of FFAs and proinflammatory cytokines, worsens insulin resistance and disease progression (60, 61) (**Figure 1**).

### 2.5 Implications for clinical management and public health

Based on the available epidemiological data, efforts aimed at early identification and management are vital in MASLD patients. Lifestyle changes like increased physical activity and alterations in diet have been found to decrease substantially the risk of developing T2DM in persons with MASLD (62). Weight loss by calorie restriction and exercise is still the most effective measure to prevent the advancement of MASLD and decrease the risk of diabetes (63). Furthermore, the available therapies aimed at the liver (steatosis, inflammation and fibrosis) are also under consideration as possible treatment options (62).

Although much remains to be understood about the association between MASLD and T2DM, some important issues include the problem of early diagnosis and risk assessment (64). MASLD does not have standard screening recommendations for at-risk populations, thus many cases remain undiagnosed and treated only after a lapse. Moreover, new imaging techniques including transient elastography and magnetic resonance imaging-proton density fat fraction (MRI-PDFF) have enhanced the diagnostic capacity but they are not yet widely available for use (65, 66). Numerous studies have established that MASLD is a significant precursor to T2DM, contributing to the development and progression of insulin resistance and glucose dysregulation (23, 34, 40). The evidence presented in this review further supports the role of MASLD in driving metabolic dysfunction, highlighting the need for large-scale, longitudinal studies to confirm its causal impact on T2DM. As the prevalence of both MASLD and T2DM continues to rise, a proactive and multidisciplinary approach involving hepatologists, endocrinologists, and public health specialists will be essential in developing effective preventive and therapeutic strategies to mitigate the burden of these interconnected diseases.

## 3 Diagnosis

MASLD is increasingly considered a significant problem in people with T2DM owing to the increasing evidence that links these two conditions. The prevalence of MASLD is 60% to 70% among patients with T2DM (67). Hence, it is important to recognize MASLD in order to manage the disease effectively. The European Association for the Study of Diabetes (EASD) and the European Association for the Study of Obesity (EASO) have highlighted the need for MASLD screening in patients with T2DM (68). The American Association for the Study of Liver Diseases (AASLD) has provided recommendations for the identification of people who are at high risk of having MASLD and advanced fibrosis and these include obese, metabolic syndrome, and T2DM patients (69).

MASLD can also develop in people with lower BMI. The threshold for defining MASLD as having a BMI <25 kg/m² depends on the non-Asian populations; in Asians it is <23 kg/m². Patients with low BMI but with metabolic complications such as T2DM, dyslipidemia or hypertension are also prone to develop MASLD (17). MASLD cannot be diagnosed before eliminating secondary causes of liver fat accumulation and significant alcohol consumption (more than 21 drinks/week for men and 14 drinks/week for women) (70).

The diagnosis of MASLD is important at an early stage in patients with T2DM in order to determine those who are likely to have progressive liver diseases. Several techniques based on imaging, genetics, and biochemistry are available for the diagnosis. Each method has its advantages and limitations. Before starting the MASLD diagnostic algorithm, liver steatosis and fibrosis must be excluded from other causes, including alcohol abuse, use of certain drugs, hereditary diseases, exposure to environmental toxins, other gastrointestinal diseases, and other conditions, including chronic HCV infections, polycystic ovary syndrome, hypothyroidism, and amphetamine use (71–73).

### 3.1 Imaging studies

Noninvasive scoring systems are increasingly important for the early detection of advanced fibrosis in MASLD. Among them, the FIB-4 index—which incorporates age, aspartate aminotransferase, alanine aminotransferase, and platelet count—is the most widely validated. A FIB-4 score <1.3 effectively rules out advanced fibrosis, while a score >2.67 rules it in, providing a simple, low-cost screening tool that can be applied in both primary care and specialist settings (74–76). Sequential algorithms combining FIB-4 with imaging modalities such as transient elastography (FibroScan) enhance diagnostic accuracy and reduce unnecessary referrals. Incorporating such noninvasive tools into routine screening is critical for identifying at-risk MASLD patients with T2DM before progression to advanced liver disease (69).

Imaging studies are essential in the diagnosis of MASLD and include non-invasive means of determining the level of liver fat and other associated abnormalities. They enable the identification of hepatic steatosis and to differentiate between MASLD and other liver diseases (77, 78). The main imaging techniques used in the diagnosis of MASLD are ultrasound, magnetic resonance imaging (MRI), computed tomography (CT), and transient elastography (TE), all of which are different in their advantages and limitations.

#### 3.1.1 Ultrasound

Widely available and cost-effective but limited by operator dependence and poor sensitivity for mild steatosis. Ultrasound is often used to diagnose MASLD and its progression to T2DM. It can detect liver fat content and fibrosis directly, which are the most important predictors of MASLD’s development to T2DM. As a non-invasive, easily accessible, and relatively cheap imaging method, ultrasound is usually used for the initial assessment of MASLD, especially in middle-aged men (78). It does not expose patients to radiation.

The development of ultrasounds has improved the use of ultrasound in the management of MASLD. Techniques that include 2D-SWE and UGAP improve the sensitivity and specificity of liver disease staging. These modalities are useful in evaluating liver stiffness and fat content to help in the diagnosis of MASLD and tracking the disease to fibrosis and cirrhosis, respectively (79).

USAT is another technique that determines the sound-energy loss through the liver based on ultrasound attenuation. Relative to CAP, USAT has a high sensitivity and specificity in the assessment of the grades of hepatic steatosis with the optimal cut off points for each stage of steatosis (80).

The application of deep learning algorithms in ultrasound imaging has also increased the sensitivity and specificity of the diagnosis, as well as reducing the possibility of the operator’s bias (81). The diagnosis of MASLD by ultrasound is in accordance with histological diagnosis of NASH and ALT levels, thus confirming the efficacy of ultrasound in clinical practice (82).

However, there are some disadvantages of using ultrasound. It has a low sensitivity to mild steatosis and the performance is dependent on the operator which can be suboptimal in some clinical situations.

#### 3.1.2 MRI

Magnetic Resonance Imaging (MRI) is now widely used in the diagnosis of MASLD as it is a non-invasive, reproducible and accurate measurement of hepatic steatosis. Highly accurate and reproducible, with strong correlation to histology, but constrained by high cost and limited accessibility. It can not only assess the fat content in the liver but also the stiffness of the liver and iron content, which gives an overall picture of the liver condition (78). The performance of MRI in diagnosing liver diseases has been enhanced by sophisticated imaging modalities, including Magnetic Resonance Elastography (MRE) and T1 mapping (83).

Specifically, MRI is very good at measuring the liver fat content. Therefore, MRI-based techniques, including Proton Density Fat Fraction (PDFF) and MRI Spectroscopy, have been found to be accurate in measuring liver fat even in low levels of fat accumulation. These methods provide a non-biopsy alternative to the conventional liver biopsy (84, 85). PDFF is a form of MRI that measures the fat content of the liver by determining the chemical shift between fat and water and may be a biomarker for MASLD. It accurately classifies the hepatic steatosis and the concordance with histological findings is good, thus attesting to its credibility as a diagnostic tool. MRI-PDFF has been found to have a high correlation with the percentage of steatotic hepatocytes and may be used as a non-invasive alternative to liver biopsy (85–87).

The use of MRI spectroscopy helps in the assessment of the fatty infiltration of the liver through the fat to water ratio of the liver, and therefore it is a specific and precise method of diagnosis of MASLD especially in patients without diabetes (85). Other MRI sequences including out of phase, in phase, and diffusion weighted imaging also help in the evaluation of fat and water content of the liver and thus give further information on the liver status (88). The precision of the MRI in identifying MASLD among individuals with obesity and metabolic syndrome has been enhanced by the recent developments in the MRI technology.

#### 3.1.3 CT scan

CT is a non-invasive method of diagnosing hepatic steatosis, and though it is not the primary method because of the concerns about radiation danger, it is useful for determining disease severity, especially in cases where other imaging methods, such as ultrasound or MRI, are uninformative (89, 90). Sensitive for moderate steatosis and useful when MRI or ultrasound are inconclusive, though radiation exposure and lower sensitivity for mild disease limit its clinical utility. CT scan findings, when used with other imaging techniques including MRI and ultrasound, increase the accuracy of the diagnosis of MASLD. Using CT in association with these techniques has been found to enhance the diagnostic assessment of fatty liver disease (91). Non-contrast CT is very sensitive and specific in the diagnosis of moderate hepatic steatosis, and Hounsfield unit (HU) values are used in the assessment of liver fat. The CT based liver density is related to anthropometric metabolic risk markers like waist circumference and subcutaneous fat thickness (92, 93).

Of the various techniques, the texture-based classification of CT images holds promise for distinguishing between benign and metastatic liver lesions (94). These methods may offer a better diagnostic potential for several liver diseases including MASLD.

#### 3.1.4 Transient elastography (FibroScan)

Transient elastography (TE), also known as FibroScan, is a non-invasive method of fibrosis and steatosis liver grading. Reliable for fibrosis staging and steatosis quantification, but performance declines in obese patients and remains influenced by operator experience. As liver fibrosis and steatosis are major factors leading to the development of diabetes, FibroScan is a useful tool in the management of MASLD patients as it gives information on the progression of the disease (95). FibroScan is a less invasive method of liver biopsy, which has its own set of complications. TE works by measuring liver stiffness; hence, it is fairly consistent with the level of fibrosis and can be used in staging the level of liver disease. The controlled attenuation parameter (CAP) is applied specifically for the assessment of steatosis in FibroScan (96).

FibroScan is easy to use, relatively cheap and the results are instantaneous. It has been found to have high sensitivity and specificity in the diagnosis of advanced fibrosis and cirrhosis, and the findings have high agreement with the histological findings (97). CAP is most important in the grading of hepatic steatosis and gives accurate results at all levels of fatness (95).

Fibrosis assessment by FibroScan has been found to be associated with increased risk of cardiovascular complications in MASLD patients, which points to the possibility of fibrosis as a marker of cardiovascular risk in these patients (98). Nevertheless, there are some disadvantages of this method including the impact of obesity and the experience of the operator. FibroScan data combined with clinical parameters may increase the diagnostic value and prognostic stratification of MASLD patients (99).

#### 3.1.5 Xenon-133 liver scan

Xenon-133 liver scan is a non-invasive imaging method that uses a radioactive gas (xenon) to diagnose hepatic steatosis or fatty liver disease. Offers non-invasive fat quantification but is not widely available and remains largely experimental compared with MRI or FibroScan. This technique looks at hepatic fat and is a way of avoiding liver biopsy, which is expensive and invasive. This imaging method detects MASLD cases early to treat them before they progress to T2DM (100). Thus, imaging techniques are important in the diagnosis and management of MASLD and the primary imaging techniques are MRI and ultrasound. These imaging modalities have their own advantages and can be used together for better diagnostic accuracy (101). However, major technical and logical difficulties, such as cost, availability, and the need for further work on new approaches, are still present. With the growing understanding of MASLD and ongoing research, it is anticipated that the integration of artificial intelligence and non-invasive biomarkers will enhance the development of diagnostic criteria for MASLD in the future (102, 103).

### 3.2 Combination of imaging and biomarkers

No single imaging technique has to date shown high specificity for MASLD. Imagers are often used however in conjunction with these scales. They are of different sensitivity and specificity but their use along with imaging techniques can improve the accuracy of MASLD diagnosis to offer a safer and noninvasive option to patients. The sensitivity and specificity of the diagnostic methods of MASLD may alter the definition of MASLD and its coexistence with T2DM (104).

Imaging and Biomarkers are combined for optimal risk stratification and treatment decision making. For instance, VCTE can help in the identification of patients with advanced fibrosis who may require more intense management. The potential for enhancing the accuracy can be explored through the combination of VCTE with serum biomarkers. However, these tools may not explain all the mechanisms that lead to the evolution of MASLD to T2DM, and other mechanisms may remain unidentified (105). However, the implementation of non-invasive scoring systems in clinical practice has given patients and clinicians an easy and convenient way of managing MASLD.

### 3.4 Biochemical and laboratory diagnostics

Biochemical and laboratory diagnostics are essential in understanding the progression from MASLD to T2DM. Liver enzymes, markers of glycemic control, markers of insulin resistance and advanced biomarkers help to reveal the pathophysiology of MASLD and its progression to T2DM (106). The use of clinical and biochemical models with LFTs, glycemic markers and insulin resistance markers increases the sensitivity of T2DM onset in MASLD patients (107). Because these markers are closely related to insulin resistance, which is a key factor in the development of T2DM, they are useful for early diagnosis and management of MASLD (8).

#### 3.4.1 Liver function tests

Liver function tests (LFTs) are very useful in diagnosing MASLD and in staging the severity of the liver disease leading to T2DM. Liver enzymes like alanine transaminase (ALT) and aspartate transaminase (AST) are usually used in LFTs to check for liver functions. These enzymes are raised in MASLD patients and this indicates that the liver is damaged or inflamed and this is associated with insulin resistance which is a key factor in the development of type 2 diabetes (8). Although liver biopsy is the most reliable method of diagnosing MASLD, LFTs are used frequently for screening purposes (108).

Serum bilirubin, transaminases, alkaline phosphatase, and gamma-glutamyl transferase are the LFTs that are used as non-invasive measures of liver disease severity and progression. A new LFT score has been shown to be accurate in categorizing the metabolic associated steatotic liver disease (MASLD) patients into cholestatic, mixed or hepatocellular subtype to help with prognostic assessments (109). In primary care settings, intelligent Liver Function Testing (iLFT) automated pathways enhance the diagnostic precision and patient care by following up the abnormal LFT results (110).

MASLD, serological index testing and LFTs are used in the diagnosis and extent of the disease respectively. However, it is important to note that LFTs measure liver enzymes which are indicators of liver injury and not liver function (111). LFTs alone are not enough to fully determine the state of the liver, other diagnostic methods must be employed as well (112).

#### 3.4.2 Glycemic control indicators

MASLD can lead to more severe hepatic diseases and is closely linked to T2DM, especially with poor glycemic control, which worsens the liver injury. Fasting plasma glucose, HbA1c levels and the OGTT are important glycemic control measures that offer valuable information on glucose metabolism and the degree of liver injury that are useful in the management and possibly the prevention of progression from MASLD to T2DM (1, 113).

HbA1c is one of the most commonly used markers of long-term glycemic control, which represents the average blood glucose levels over the past 2–3 months. It has some advantages for diabetes management since it is logistically simple – fasting is not required (114). Previous research has indicated that higher HbA1c levels are linked with more severe MASLD, including fibrosing liver disease and NASH (115, 116). Fasting plasma glucose is a conventional glycemic control marker that gives an immediate reading of the blood glucose concentration after a period of fasting. It is usually employed together with other tests to make the diagnosis. Fasting plasma glucose levels are increased in both MASLD and T2DM, suggesting a disturbance in glucose metabolism (9, 17, 18).

The OGTT is a test that helps in the assessment of the body’s capacity to break down glucose and is done to diagnose impaired glucose tolerance and type 2 diabetes (117). HbA1c, however, does not show the detailed changes in glucose metabolism during the OGTT. But because of the need to fast and draw blood several times, it is not as suitable for repeated use as a screening tool. HbA1c is the preferred method for daily clinical practice.

### 3.5 Histological evaluation

#### 3.5.1 Liver biopsy

The progression from MASLD to T2DM is a metabolic process that leads to insulin resistance and poor glycemic control (118). Liver biopsy is a diagnostic tool employed to establish the presence of MASLD and to evaluate the severity of the disease which is important in the prediction of T2DM development. Liver biopsy is however invasive and is associated with some risks (8, 9).

Liver biopsy offers a higher level of histological detail, including steatosis, inflammation and fibrosis. It is still the gold standard for the diagnosis of NASH and the assessment of fibrosis stage which are important in management of the patient and in clinical trials (119, 120). This is because non-invasive methods cannot offer the level of detail that liver biopsy offers. Also, liver biopsy is important in distinguishing between simple steatosis and NASH which is more severe and is associated with worse liver outcomes (96, 121).

The invasiveness of the procedure and the costs that come with it are barriers to its use. Inaccurate sampling of liver tissue can lead to incorrect staging of MASLD because of the heterogeneity of the liver (122). Intra and inter observer variability in the analysis of biopsy data also affects the credibility of the diagnosis. Liver biopsy is also quite costly, which creates a problem for its use in large-scale screening (123).

Liquid biopsy techniques are now available as non-invasive methods of detecting and managing liver diseases and have been shown to be accurate in the diagnosis of NASH and liver fibrosis (124). AI and advanced imaging are also being investigated for possible use instead of liver biopsy but these are not yet widely used in clinical practice. More work is still required to validate non-invasive diagnostic tools that can be used for routine screening and risk stratification of MASLD and T2DM patients.

## 4 Mechanisms linking MASLD to T2DM development

### 4.1 Activation of mTORC1

MASLD is a metabolic disorder that occurs due to augmentation of lipids in the liver leading to metabolic dysfunction. The main causes of this disease include overnutrition and higher levels of glucose and free fatty acids in the blood that stimulate insulin secretion (46). Insulin, in turn, activates the phosphoinositide 3-kinase (PI3K)-AKT signaling pathway. This pathway is also involved in the regulation of metabolism of glucose and lipids. One of the major secondary effects of AKT activation is the stimulation of the mammalian target of rapamycin complex 1 (mTORC1), a nutrient-loaded sensor complex that stimulates lipid production (125, 126).

Stimulation of mTORC1 increases the expression of SREBP-1c, a transcription factor that controls the genes that encode enzymes that facilitate *de novo* lipogenesis (DNL) (127). SREBP-1c increases the expression of ACC and FAS, the enzymes that catalyze the synthesis of fatty acids and, in turn, TGs in hepatocytes (128, 129). Though the liver stores lipids in the form of TG, excess lipids also generate toxic lipid species like DAGs and ceramides. These lipid intermediates act as activators of PKCϵ, which in turn dephosphorylates AKT and disrupts insulin signaling and thus leads to insulin resistance (130, 131).

As insulin resistance develops, the liver becomes less sensitive to insulin, which decreases glucose uptake and increases glucose production by the liver, which in turn worsens hyperglycemia (118). In an attempt to compensate for this metabolic dysfunction, the pancreas secretes more insulin; however, as the insulin resistance progresses, the pancreatic β-cells are damaged and contribute to the pathogenesis of T2DM (118, 132). In addition, lipid accumulation and insulin resistance can lead to the development of hepatic inflammation and fibrosis and thus increase the risk of progression to NASH and cirrhosis, respectively (133). Disrupted lipid metabolism in MASLD plays a key role in driving systemic metabolic complications, leading to the progression from hepatic steatosis to insulin resistance and ultimately T2DM.

### 4.2 Insulin resistance

IR is the primary factor leading to the development of MASLD and its progression to T2DM. Metabolism plays a significant role in the development of IR, as metabolic dysfunction worsens IR and disease progression (134).

MASLD is characterized by the accumulation of lipids in the liver through intrahepatic TG. The increase in intrahepatic TG content is attributed to increased lipid synthesis, including DNL and increased hepatic uptake of FFAs from adipose tissue. Also, reduced hepatic TG catabolism by decreased β-oxidation and decreased very low-density lipoprotein (VLDL) export adds to the accumulation of lipids in the liver (135, 136).

IR affects metabolic homeostasis by increasing DNL in the liver and decreasing the ability of adipose tissues to suppress lipolysis. DNL is increased in MASLD and is a key factor in the development of this disease and is increased in liver of patients with IR by the activation of SREBP-1c (137). This leads to increased TG synthesis and storage and hence aggravates lipid accumulation and liver injury. Steatosis of the liver occurs when the fat content of the liver exceeds its ability to remove fat (138, 139).

Oxidative stress, inflammation, ER stress, overnutrition, and obesity have been identified to stimulate SREBP-1c (140). The farnesoid X receptor (FXR), a nuclear receptor, exerts a negative control on DNL by interfering with SREBP-1c (141). It has been observed that mice with obesity induced by high fat diet are deficient in FXR expression. FXR knockout leads to hepato-steatosis whereas overexpression of FXR reverses hepatic steatosis (142, 143). Yin Yang 1 has been postulated to act as an inhibitor of FXR transcription in obese mice (144). Thus, the role of FXR deficiency in the pathogenesis of MASLD and its progression to T2DM cannot be overemphasized.

The main mechanisms of pathogenesis of T2DM are IR in peripheral tissues and β cell failure in the pancreas (145). Glucose homeostasis is regulated by the liver through hepatic glucose production (HGP) and glucose utilization, which are both regulated by the concentration of glucose in the blood (146). Fasting hyperglycemia, a defining feature of T2DM, is caused by increased gluconeogenesis and increased hepatic glucose output. Increased levels of 17-hydroxyprogesterone (17-OHP) in MASLD is due to the aberrant expression of cytochrome P450 17A1 (CYP17A1) which stimulates gluconeogenesis through a glucocorticoid receptor-dependent pathway. This also enhances hepatic glucose production. In mouse models of diabetes, Cyp17A inhibition leads to a marked glucose lowering effect (147, 148).

A study reported that chronic low-grade ER stress in the livers of obese MASLD mice stimulates gluconeogenesis by stabilizing cyclic AMP-responsive element-binding protein (CREB) through ubiquitin-specific peptidase 14 (USP14) activation (149). MASLD boosts gluconeogenesis through several mechanisms. The hepatokines including fetuin-A participate in the regulation of insulin sensitivity and glucose metabolism. Fetuin-A acts as an endogenous TLR4 ligand and thereby induces inflammation and insulin resistance in adipose tissue (150). Another study showed that the liver enzyme FBXW7 targets fetuin-A for degradation by the proteasome. In MASLD, FBXW7 down-regulation leads to the enhancement of fetuin-A, which in turn increases systemic insulin resistance (151). Periostin, another hepatokine that is increased in MASLD, also contributes to hepatic insulin resistance through the JNK/c-Jun signaling pathway and suppression of fatty acid oxidation (152).

Hyperglycemia is also worsened by the hepatic insulin resistance that results in failure of gluconeogenesis suppression and increased hepatic glucose production, a key event in the pathogenesis of T2DM (132, 153–155). Hepatic steatosis is characterized by the perpetuation of hepatic DNL even in the condition of insulin resistance, which in turn leads to hepatic steatosis and metabolic deregulation. The disturbances in glucose and lipid metabolism in the liver lead to hyperglycemia and hyperlipidemia, thereby forming the basis of MASLD progression to T2DM (153, 156).

Normally, insulin suppresses gluconeogenesis and enhances glycogen synthesis. However, in states of insulin resistance, these processes are inverted, and there is paradoxical increase in gluconeogenesis and lipogenesis (157). The transcription factor CREB, in association with its coactivator CRTC2, is involved in the regulation of gluconeogenic gene expression. Proteins such as Sam68 increase this process and thus worsen IR and glucose dysregulation (158, 159).

Lipid accumulation in hepatocytes also causes ER stress and impairs insulin signaling and enhances hepatic inflammation (160). Hepatic insulin resistance and MASLD are connected to adipose tissue dysfunction, including adipo-IR. Enhanced lipolysis in the adipose tissues of insulin resistant patients enhances the influx of FFAs to the liver which in turn worsens hepatic steatosis and insulin resistance. IR in the skeletal muscle and other peripheral tissues also adds to the systemic metabolic abnormalities and the progression to T2DM (161).

Critical among the pathways implicated in the development of hepatic IR are the insulin signaling pathways involving IRS-1/2 and PI3K/Akt. These pathways control metabolic functions of insulin and their altered activation results in increased gluconeogenesis and hepatic lipid accumulation (125, 162, 163). VEGFB has been found to be involved in the control of insulin resistance in MASLD through the PI3K/Akt pathway and the regulation of glucose and lipid metabolism. Overexpression of VEGFB enhances insulin sensitivity through increasing glucose uptake and suppressing gluconeogenesis. The hallmark of hepatic IR, increased gluconeogenesis, is a key event in the progression of simple steatosis to NASH and progressive fibrosis (164, 165).The TXA2 receptor (TP) stimulates hepatic IR and steatosis through the Ca2+/CaMKIIγ-PERK-CHOP-TRB3 signaling pathway (166). The TXA2/TP pathway has been found to be a potential therapeutic target to enhance insulin sensitivity and to prevent hepatic steatosis and inflammation in MASLD patients (167).

### 4.3 Lipotoxicity

The pathogenic process of lipid accumulation and resultant insulin resistance in the progression from MASLD to T2DM is complex. Lipotoxicity is the accumulation of toxic lipid species in non-adipose tissues such as the liver and is involved in insulin resistance, inflammation, and cellular stress that leads to the development of MASLD to T2DM (168, 169). An increase in plasma FFAs – mainly due to enhanced lipolysis in adipose tissue – results in increased flux of FFAs to the liver. This causes hepatic steatosis, a feature of MASLD, which in turn affects insulin signaling and thus induces insulin resistance. Thus, insulin resistance increases the severity of MASLD through the stimulation of the *de novo* lipogenesis in the liver (170). The expression of carbohydrate-responsive element-binding protein (ChREBP) is upregulated to increase the synthesis of fatty acid and TG (171). The lipotoxicity leads to the accumulation of diacylglycerol and ceramides that disturb the cell metabolism and lead to insulin resistance and β-cell dysfunction that are both critical for the development of T2DM. The accumulation of lipids in other tissues results in cellular dysfunction and hepatocyte damage. This manifests as hepatic injury and inflammation in the liver and can range from simple steatosis to metabolic dysfunction-associated steatohepatitis (MASH), also known as nonalcoholic steatohepatitis (NASH) (172, 173). The accumulation of toxic lipids also leads to the activation of ER stress, oxidative stress, and mitochondrial dysfunction. These processes lead to β-cell dysfunction in the pancreas and thereby to impaired insulin secretion and insulin resistance (174). ER stress is activated by lipotoxicity and leads to the activation of the unfolded protein response (UPR) that causes hepatocyte apoptosis and inflammation. This leads to the progression of MASLD to MASH and finally to T2DM (175). Moreover, mitochondrial dysfunction enhances the generation of reactive oxygen species (ROS), which worsen liver inflammation and β-cell damage in the context of MASLD progression to T2DM (176). Lipotoxicity also stimulates NLRP3 inflammasomes to secret proinflammatory cytokines like IL-1β that triggers liver inflammation and insulin resistance. Moreover, lipotoxicity also activates inflammatory pathways, including NF-κB, to worsen insulin resistance and β-cell dysfunction (177, 178). The upregulation of CD36/FAT translocase enhances hepatic FFA uptake, facilitating the progression of MASLD to T2DM. Genetic predisposition, particularly in lipid metabolism and insulin signaling pathways, further modulates disease susceptibility (179, 180). In the context of lipotoxicity, small extracellular vesicles (sEVs) secreted by β-cells exhibit altered protein and lipid compositions, contributing to β-cell dysfunction and apoptosis (181).

### 4.4 Role of adipokines and inflammatory mediators

Adipokines are bioactive molecules produced by adipose tissues that regulate glucose and lipid metabolism; in MASLD, they are dysregulated. These molecules are mediators in key pathways regulating metabolism and inflammation that ultimately impact insulin sensitivity and glucose homeostasis (182, 183). Chronic low-grade inflammation, driven by proinflammatory cytokines, exacerbates insulin resistance and hepatic steatosis and thus accelerates disease progression.

#### 4.4.1 Leptin and adiponectin

Leptin is a key player in the regulation of energy balance and glucose metabolism. It increases insulin sensitivity and glucose uptake in the peripheral tissues. However, in obesity, leptin resistance is developed, which leads to impaired glucose metabolism and an increased risk of developing T2DM (184). Adiponectin is another important adipokine that increases glucose metabolism by increasing the uptake of glucose and the oxidation of fatty acids. Adiponectin concentrations are inversely related to body fat and people with low levels are insulin resistant (185, 186).

Adiponectin has an anti-inflammatory action that improves insulin sensitivity. Its concentration is reduced in obesity, which leads to a pro-inflammatory state that worsens MASLD (182). However, adiponectin aids in the breaking down of lipids and reduces the accumulation of fat in the liver, thus preventing the progression of MASLD (186). Leptin levels are, however, elevated in MASLD and advocate for inflammation and insulin resistance. Leptin and adiponectin are two proteins that are usually imbalance in MASLD and this imbalance plays a role in the progression of hepatic steatosis and inflammation (187).

#### 4.4.2 Resistin and visfatin

Resistin is an adipokine that is associated with insulin resistance. At elevated levels of resistin, glucose production is increased and insulin sensitivity is reduced, thus contributing to the development of T2DM (188). Visfatin is another adipokine that is involved in lipid metabolism and promotes adipogenesis and cholesterol accumulation. Visfatin at elevated levels deteriorates MASLD and leads to insulin resistance (189). Resistin is connected with insulin resistance and inflammation, whereas visfatin is connected to fibrosis progression in MASLD (188, 189). Both adipokines are increased in MASLD patients especially those with obesity and thus play a role in disease progression.

#### 4.4.3 Chemerin and other adipokines

Chemerin increases adipocyte differentiation and lipid storage by regulating lipid metabolism, thus causing fat accumulation and insulin resistance. MASLD is associated with altered levels of adipsin and plasminogen activator inhibitor-1 (PAI-1), whereas adipsin is markedly higher in obese MASLD patients than in normal-weight individuals (190, 191). These findings are significant in highlighting the role of adipokines in the pathogenesis and progression of MASLD.

Hepatic steatosis and inflammation are classic features of insulin resistance, and the dysregulation of adipokines is implicated in their development (192). Leptin and resistin are involved in insulin resistance; however, reduced levels of adiponectin are unable to counter these effects (186). The relative levels of these adipokines are such that they adversely affect insulin sensitivity; adiponectin improves insulin signaling, while resistin blocks it, thus leading to insulin resistance and facilitating the progression from MASLD to T2DM (193).

#### 4.4.4 Role of TNF-α and IL-6

**Figure 2:**
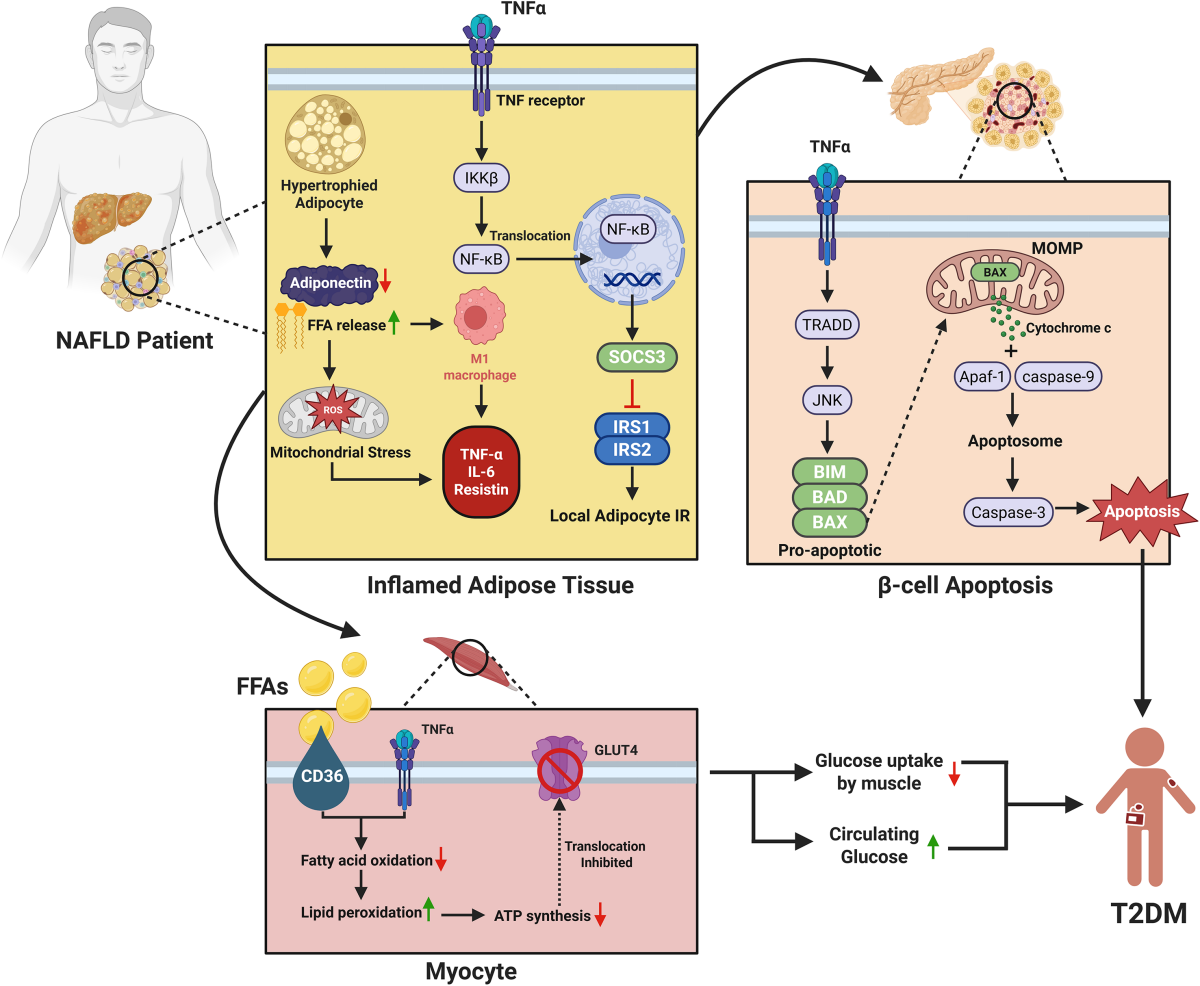
Mechanistic link between MASLD and the development of type 2 diabetes mellitus (T2DM). The figure illustrates the molecular links between MASLD and T2DM through adipose inflammation, β-cell apoptosis, and muscle insulin resistance. In MASLD, inflamed adipose tissue releases excess free fatty acids (FFAs) and pro-inflammatory cytokines (e.g., TNF-α, IL-6, resistin) while adiponectin levels decline. TNF-α activates the IKKβ/NF-κB and SOCS3 pathways, promoting insulin resistance in adipocytes. It also induces β-cell apoptosis via TRADD/JNK signaling and mitochondrial cytochrome c-mediated caspase activation, reducing insulin secretion. Simultaneously, FFAs and cytokines impair skeletal muscle insulin sensitivity by inhibiting fatty acid oxidation and the translocation of GLUT4, leading to decreased glucose uptake and contributing to systemic insulin resistance and the progression of T2DM.

Tumor necrosis factor-alpha (TNF-α) plays a critical role in the progression of MASLD and its conversion to T2DM. TNF-α activates hepatic inflammation and fibrosis and leads to severe liver and metabolic complications. The blockade of TNF-α has been linked with enhancement of hepatic damage markers and decrease in insulin resistance which supports the crucial role in the pathogenesis of MASLD (194). Genetic modulation of TNF-α in animal models has been reported to alleviate metabolic dysfunction and hepatic inflammation, thus pointing to its importance in the progression of the disease (194–196) (**Figure 2**).

Interleukin-6 (IL-6) is a cytokine with both inflammatory and metabolic regulatory roles. IL-6 also regulates mitochondrial dynamics in the liver, and therefore energy metabolism and development of T2DM. The relationship between IL-6 levels and the severity of MASLD indicates its possible use as a biomarker for disease progression and a possible treatment target (197).

MASLD is characterized by chronic low-grade inflammation, which is characterized by increased levels of TNF-α and IL-6, which are in turn associated with insulin resistance and T2DM onset (198). Oxidative stress, lipotoxicity, and adipose tissue dysfunction act synergistically to worsen the inflammatory response and the impairment of insulin signaling. The gut-liver axis and immune activation are key in the maintenance of the inflammatory state, which supports the need for specific therapeutic approaches to prevent the progression of MASLD to T2DM.

### 4.5 Translational implications of immunometabolism

The immunometabolic pathways that connect MASLD with T2DM also provide therapeutic entry points. Elevated TNF-α and IL-6, central to hepatic inflammation and systemic insulin resistance, are potential targets for anti-cytokine therapies currently evaluated in metabolic disease (199, 200). Dysregulated adipokines highlight opportunities for intervention: agents that increase adiponectin signaling may restore insulin sensitivity and reduce steatosis. Lipotoxicity-induced ER stress and SREBP1c activation are being targeted by small-molecule SREBP1c inhibitors, while FXR agonists such as obeticholic acid aim to rebalance bile acid signaling, *de novo* lipogenesis, and hepatic inflammation (186, 201, 202). Furthermore, incretin-based therapies (GLP-1 receptor agonists and dual agonists such as tirzepatide) improve hepatic steatosis, modulate inflammatory responses, and protect β-cell function, illustrating how mechanistic insights can be translated into pharmacological strategies (203–205). Anchoring mechanistic pathways to emerging therapies underscores the clinical relevance of immunometabolic dysregulation in MASLD–T2DM.

### 4.6 Role of hepatic macrophages

**Figure 3:**
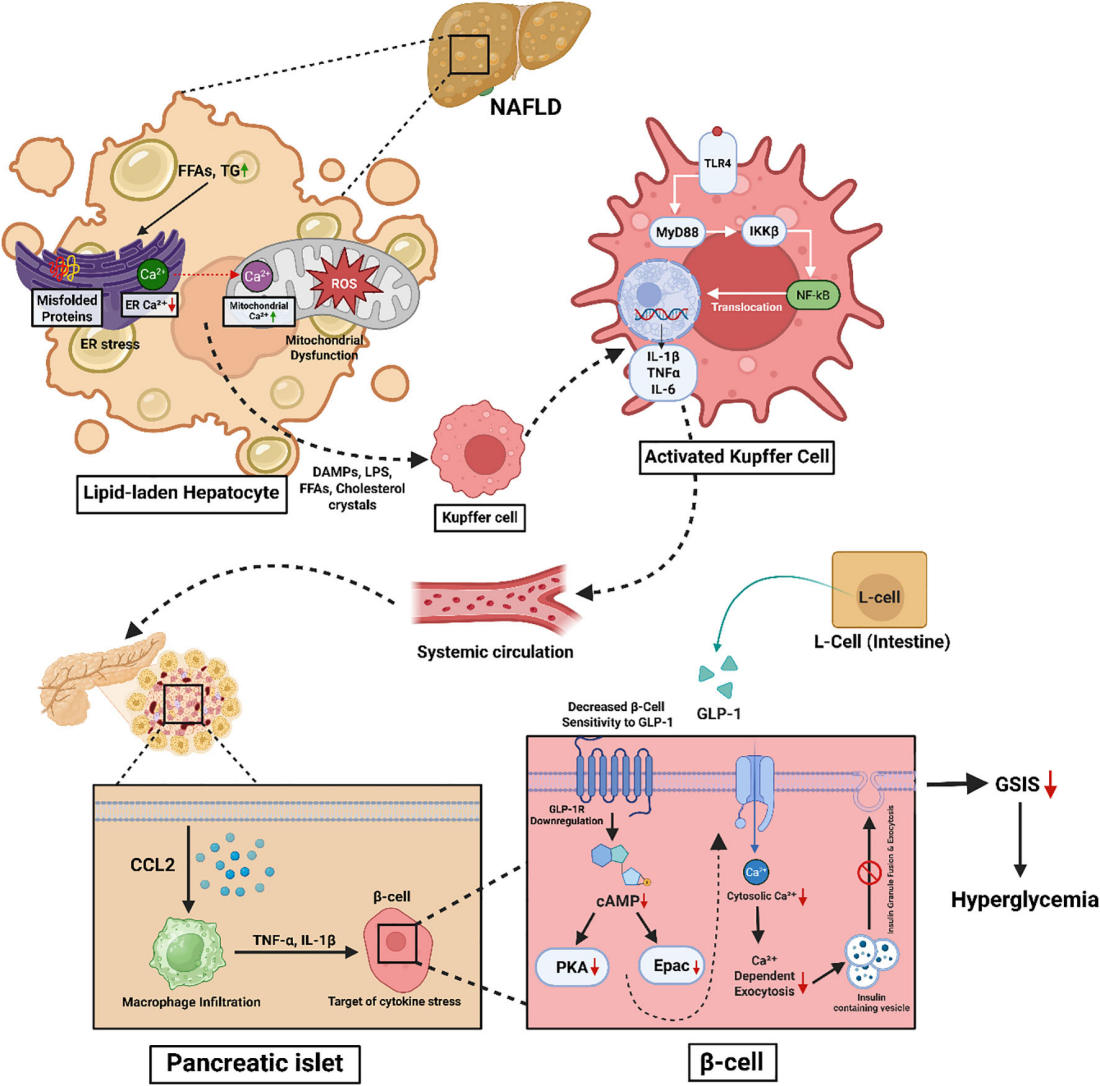
Schematic representation of the mechanistic link between MASLD-induced hepatic stress and pancreatic β-cell dysfunction contributing to hyperglycemia. In MASLD, lipid accumulation in hepatocytes leads to ER stress, mitochondrial dysfunction, and ROS production. These stress responses trigger the release of DAMPs, LPS, FFAs, and cholesterol crystals, activating Kupffer cells via the TLR4–MyD88–NF-κB pathway. Activated Kupffer cells secrete pro-inflammatory cytokines (IL-1β, TNF-α, IL-6), which circulate systemically and induce inflammation in pancreatic islets. Infiltrating macrophages in the islets release cytokines (e.g., TNF-α, IL-1β), impairing β-cell function. Concurrently, intestinal L-cell-derived GLP-1 signaling is blunted due to GLP-1 receptor downregulation and reduced cAMP signaling in β-cells, leading to impaired Ca²^+^-dependent insulin exocytosis. The combined effects result in diminished glucose-stimulated insulin secretion (GSIS) and contribute to hyperglycemia.

The level of hepatic macrophage infiltration is a key determinant of the severity of MASLD. Macrophages activate and sustain both local and systemic inflammation that underlies insulin resistance, a well-known cause of T2DM. The mechanism is complex and involves various macrophage subsets, inflammatory mediators, and metabolic pathways (206) (**Figure 3**).

Kupffer cells (KCs) and monocytes-derived macrophages (MoMFs) are the primary macrophages involved in the inflammatory process of MASLD. These macrophages are in contact with hepatocytes and other liver cells, participating in inflammation and fibrosis (207). Hepatic macrophages exhibit either pro-inflammatory (M1) or anti-inflammatory (M2) phenotypes, playing a crucial role in regulating liver inflammation. M1 macrophages secrete pro-inflammatory cytokines, which contribute to hepatic inflammation and fibrosis, thereby exacerbating the progression of MASLD. A higher M1/M2 macrophage ratio in adipose tissue and the liver is associated with increased inflammatory responses, worsening insulin resistance and MASLD severity (208). This imbalance in macrophage polarization underscores the inflammatory mechanisms driving hepatic metabolic dysfunction and highlights a potential therapeutic target for mitigating MASLD-associated insulin resistance (209).

Cytokines and chemokines that are secreted by macrophages regulate the inflammatory response, which is linked with insulin resistance. Pro-inflammatory cytokines released by these macrophages disrupt insulin signal transduction, leading to systemic metabolic dysregulation. Additionally, macrophage accumulation in adipose tissue exacerbates paracrine inflammation, further impairing insulin sensitivity. Dysfunctional adipose tissue macrophages amplify systemic inflammation, which, in turn, aggravates hepatic inflammation (207). Hepatic macrophages are in contact with hepatocytes, hepatic stellate cells and endothelial cells to control the function and the pathogenesis of the liver. This dynamic is important in the maintenance of the inflammatory milieu that is characteristic of MASLD and its progression.

### 4.7 Oxidative stress

MASLD-induced lipid accumulation leads to an increase in fatty acid oxidation and the production of excessive ROS. Mitochondrial dysfunction participates in oxidative stress, which, in turn, affects ATP production and leads to hepatocyte injury. ER stress, dietary factors, and inflammation increase oxidative damage (210, 211).

Oxidative stress activates JNK and PKC signaling pathways which leads to serine phosphorylation of IRS1 and IRS2 and thereby impair insulin signaling and glucose uptake. This increase in ROS affects the activity of PI3K, which leads to the activation of NADPH oxidase 4 (NOX4) and increased production of ROS. This process also enhances the degradation of GLUT4 and hence reduces the glucose transport and increases hyperglycemia (212). The ROS accumulated in the cells secrete proinflammatory cytokines, which in turn activate NF-κB and JNK pathways and worsen the insulin signaling. Oxidative stress, being chronic in nature, affects the β cells of the pancreas and leads to β cell exhaustion and decreased insulin secretion (213, 214). The cumulative effect of oxidative stress, insulin resistance and inflammation play the key role in pathogenesis of T2DM.

The mechanisms of the pathophysiological relationships between MASLD and T2DM are complex and include lipid accumulation in the liver, insulin resistance, inflammation, altered microbiota in the gut and oxidative stress. These pathways are interlinked to form a positive feedback loop of metabolic abnormalities that predisposes to the development of T2DM. The mTORC1 activation, defective insulin signaling, lipotoxicity, cytokine production, dysbiosis of the gut and oxidative stress are the main mechanisms of MASLD complications leading to T2DM. Knowledge of these mechanisms is important in developing new strategies for treating disease progression and patient care.

The gut-liver axis is also involved in the propagation of systemic inflammation. When there is an imbalance in the gut microbiota and increased gut permeability, bacterial products are able to stimulate liver macrophages and thus perpetuate inflammation. This mechanism links MASLD with insulin resistance and T2DM (215). The majority of MASLD patients have hepatic insulin resistance and, therefore, have increased hepatic glucose production and elevated blood glucose, which further supports the pathophysiological relationship between the two conditions, MASLD and T2DM.

### 4.8 Gut microbiota dysbiosis

Dysbiosis of the gut microbiota is a typical feature of MASLD, which is characterized by changes in the microbial community. MASLD patients have decreased concentrations of good bacteria, such as F. prausnitzii, and increased concentrations of pathogenic bacteria, including E. coli. It results in the decrease of Bacteroidetes and the increase of Firmicutes and Proteobacteria (216).

The intestinal barrier is disrupted resulting in enhanced permeability also referred to as ‘leaky gut’. Lack of butyrate is known to compromise the integrity of the intestinal barrier, thus allowing bacterial endotoxins like LPS to circulate systemically. In addition, LPS coupled with metabolic endotoxemia, acts through TLR4 to induce systemic inflammation and IR (217).

Furthermore, the gut microbiota plays a role in the regulation of bile acid metabolism. Bile acids are produced in the liver and then altered to become secondary bile acids in the gut (216). MASLD is also characterized by altered bile acid metabolism that results in increased lipogenesis, IR, and inflammation. These disturbances are responsible for metabolic dysregulation, which in turn contributes to the association between MASLD and T2DM.

### 4.9 The gut–liver–brain axis

The gut–liver–brain axis is a complex, bidirectional communication network that plays a pivotal role in regulating metabolic homeostasis and is increasingly recognized as a central player in the pathogenesis of metabolic disorders and obesity (218–220). This axis comprises neural, hormonal, immune, and microbial signaling pathways that interconnect the gastrointestinal tract, liver, and central nervous system (CNS).

A key initiating factor in the disruption of this axis is “leaky gut,” which allows the translocation of microbial-derived products, such as LPS, trimethylamine-N-oxide (TMAO), ethanol, bile acids, and short-chain fatty acids (SCFAs), into the portal circulation (221). These microbial products reach the liver and activate hepatic immune cells, particularly Kupffer cells, via Toll-like receptor 4 (TLR4) signaling, triggering the release of pro-inflammatory cytokines such as TNF-α, IL-1β, and IL-6 (222). These pro-inflammatory cytokines promote hepatic environment dysfunction, leading to mitochondrial dysfunction, oxidative stress, and endoplasmic reticulum (ER) stress, ultimately impairing insulin receptor substrate (IRS) signaling (140, 223).

In parallel, these peripheral inflammatory signals and microbial metabolites can breach the blood–brain barrier (BBB) or activate afferent vagal pathways, leading to neuroinflammation in metabolic brain centers such as the hypothalamus and nucleus tractus solitarius (NTS) (224). This neuroinflammation, characterized by microgliosis and astrogliosis, disrupts central insulin signaling, thereby impairing the brain’s regulatory control over hepatic glucose production and energy balance (73, 225, 226). Consequently, endogenous glucose production (EGP) increases, exacerbating hyperglycemia and insulin resistance.

Furthermore, diminished vagal tone in metabolic disease blunts feedback regulation between the liver and brain, further perpetuating this dysfunction (227, 228). Therapeutic strategies targeting this axis—such as microbiota modulation through probiotics and fecal transplantation, enhancement of gut barrier integrity, anti-inflammatory interventions targeting cytokine pathways or TLRs, and neuromodulatory approaches like vagal nerve stimulation—offer promising avenues for restoring systemic metabolic balance (229–232). Overall, the gut–liver–brain axis represents a critical, integrated system whose disruption significantly contributes to the development of metabolic diseases, and whose restoration holds potential for novel, multi-organ therapeutic interventions.

### 4.10 Genetic and epigenetic modification

Genetic and epigenetic changes are involved in the development of MASLD by influencing the interaction between metabolic pathways, genetic factors, and environmental influences. Epigenetic alterations, which regulate gene expression without modifying the DNA sequence, play a crucial role in disease progression. These include DNA methylation, histone modifications, and non-coding RNAs, which modulate key processes such as lipid metabolism, insulin signaling, and inflammation. Understanding these regulatory mechanisms provides insight into the molecular basis of MASLD-associated metabolic dysfunction and offers potential therapeutic targets for preventing disease progression to T2DM (233).

DNA methylation is the process of chemically modifying DNA by the addition of a methyl group and it affects gene expression. In MASLD, abnormal DNA methylation affects genes that are involved in lipid metabolism and inflammation leading to the progression of the disease. Similarly, in T2DM, DNA methylation affects insulin gene expression and beta cell differentiation, glucose metabolism and insulin resistance (234). Histone modifications are variations in histone proteins that result in changes in chromatin structure and gene transcription (159, 235). microRNAs and long non-coding RNAs work post-transcriptionally to regulate gene expression. They are involved in metabolic pathways that are associated with MASLD and inflammatory signaling. Also, miRNAs regulate insulin sensitivity and beta cell function in T2DM (236, 237).

This includes long-term consumption of a high-fat diet and a lack of physical activity, which can lead to MASLD and T2DM. These factors induce epigenetic changes that exacerbate the severity of liver injury and insulin resistance. The aforementioned genetic predispositions are polymorphisms in the TET protein family, which lead to epigenetic markers such as 5-hydroxymethylcytosine that adversely affect liver mitochondrial function and therefore increase the risk of MASLD and T2DM.

Out of all the genes associated with MASLD, the most commonly examined is the I148M mutation in PNPLA3. This variant increases the liver fat content and is a marker of the progression of MASLD to more advanced liver diseases and metabolic complications and T2DM (235, 238). For instance, the TM6SF2 gene mutation E167K is associated with higher hepatic fat content but does not lead to insulin resistance or T2DM. However, this variant can worsen the liver disease and thus increase the risk of T2DM development (239). Mutations in the GCKR and MBOAT7 genes are also linked to MASLD. These genetic alterations are involved in the regulation of lipid metabolism and insulin sensitivity (240).

The PGC-1α Gly482Ser polymorphism is related with the higher susceptibility to NAFLD in the patients suffering from T2DM, probably by the impaired transcription of the PEPCK-C gene which aggravate the fat deposition (241). Among the 115 genes identified by a genome-wide study as being associated with MASLD and T2DM, 55 genes were involved in inflammation and lipid metabolism. This genetic overlap suggests that these diseases may have a similar pathogenic mechanism (242). Moreover, the differential gene expression analysis revealed 15 critical genes that are specifically associated with MASLD and T2DM, which suggests that they may have a similar pathophysiology (243).

**Figure 4:**
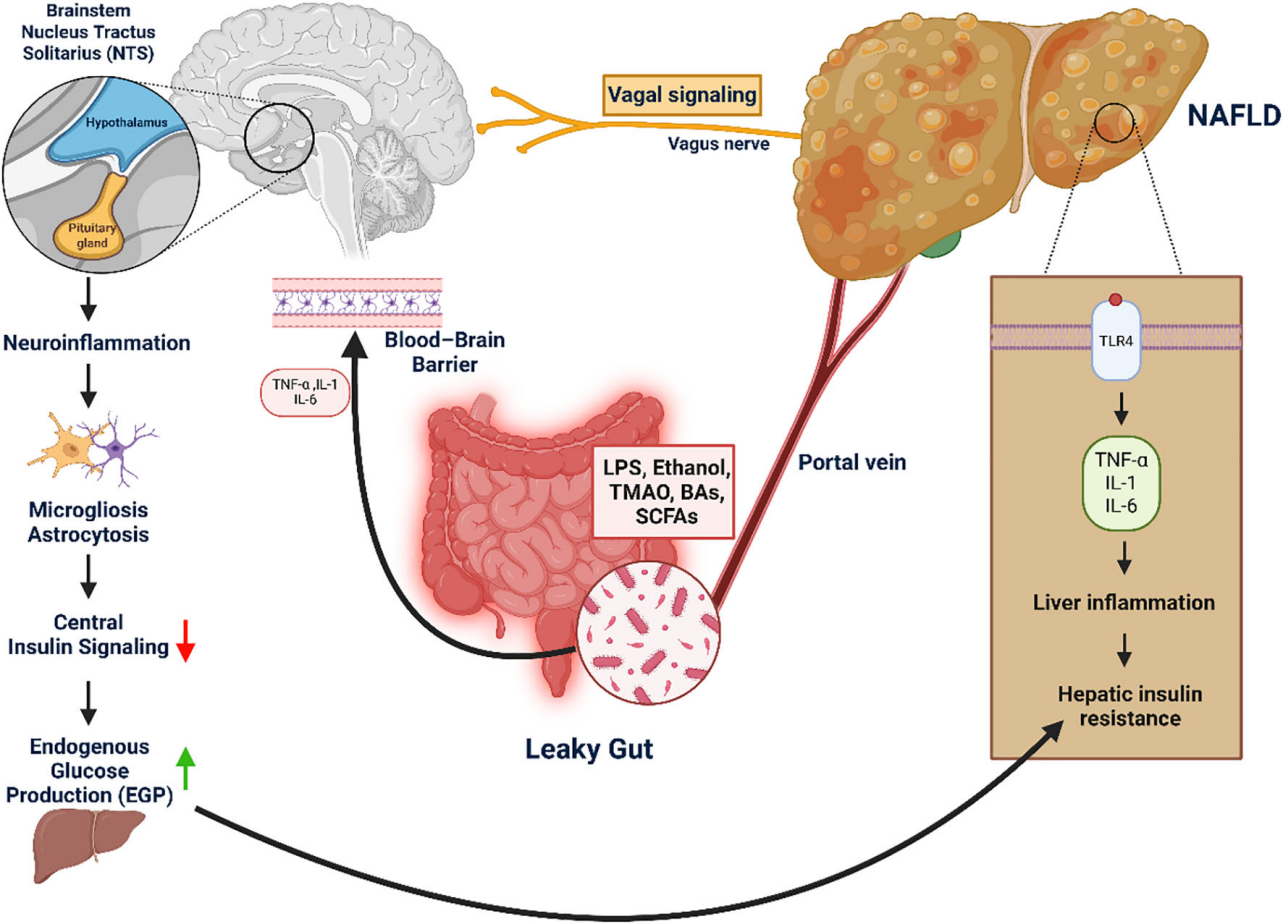
Gut–liver–brain axis in NAFLD-induced hepatic and central insulin resistance. This figure highlights the role of the gut–liver–brain axis in driving hepatic and central insulin resistance, key contributors to metabolic dysfunction. Disruption of the intestinal barrier (“leaky gut”) enables microbial products—such as LPS, ethanol, TMAO, bile acids, and SCFAs—to enter the portal circulation and reach the liver. There, they activate TLR4 signaling, triggering the release of pro-inflammatory cytokines (TNF-α, IL-1β, IL-6) that promote hepatic insulin resistance, a defining feature of MASLD. Simultaneously, systemic inflammation and gut-derived signals impair the blood–brain barrier, allowing cytokines and microbial metabolites to induce neuroinflammation in metabolic brain regions like the hypothalamus and nucleus tractus solitarius (NTS). This inflammation disrupts central insulin signaling, reducing the brain’s control over hepatic glucose output and exacerbating glucose dysregulation. Additionally, impaired vagal feedback from the liver may reinforce these disturbances in energy homeostasis.

Genetic and epigenetic variants shape susceptibility by converging on lipid handling, insulin signaling, and inflammation. The PNPLA3 I148M mutation impairs triglyceride mobilization in hepatocytes, leading to lipid retention and hepatic insulin resistance. TM6SF2 E167K reduces very-low-density lipoprotein (VLDL) secretion, exacerbating steatosis and fibrosis risk (244, 245). MBOAT7 variants alter phospholipid remodeling, thereby amplifying inflammatory signaling cascades (246). GCKR polymorphisms deregulate glucose and lipid flux, predisposing to combined dysglycemia and steatosis. Epigenetic alterations, including DNA methylation of insulin signaling genes and non-coding RNA regulation of β-cell function, further integrate environmental factors such as high-fat diets with heritable risk (247, 248). Collectively, these mechanisms explain how population-level genetic associations translate into molecular drivers of MASLD and its progression to T2DM (**Figure 4**).

## 5 Summary and conclusion

This review focuses on MASLD, previously referred to as NAFLD, as one of the most important causes of chronic liver disease worldwide, especially in relation to T2DM. Hepatic IR, lipid accumulation, and chronic low-grade inflammation primarily drive the pathophysiological connection between MASLD and T2DM. The bidirectional relationship be-tween these conditions is well established, as MASLD increases the risk of T2DM onset and progression, while T2DM exacerbates MASLD pathogenesis. The rising global incidence of MASLD parallels the increasing prevalence of obesity and metabolic syndrome, making it a major public health concern.

Recent studies indicate that over 60% of individuals with T2DM also have MASLD, emphasizing the need for early screening and intervention. The diagnosis of MASLD has shifted towards non-invasive approaches, including ultrasound, MRI, CT scans, FibroScan (transient elastography), and Xenon-133 liver scans, in combination with biochemical markers such as liver function tests (LFTs) and glycemic control measures. These methods have largely replaced liver biopsy, enhancing diagnostic accuracy while reducing patient burden.

Multiple molecular and genetic mechanisms influence the progression of MASLD in individuals with T2DM, including hepatic IR, lipotoxicity, oxidative stress, and inflammatory pathways mediated by adipokines and cytokines (e.g., TNF-α, IL-6). Genetic and epigenetic modifications, such as DNA methylation, histone modifications, and microRNAs, further contribute to disease pathogenesis. Variants in PNPLA3, TM6SF2, and GCKR genes have been identified as key genetic risk factors for both MASLD and T2DM.

Additionally, hepatic macrophage activation, gut microbiota dysbiosis, and systemic inflammation worsen IR and metabolic dysfunction. These pathological mechanisms are further aggravated by disruptions in lipid metabolism, mitochondrial dysfunction, and endoplasmic reticulum (ER) stress, collectively compromising glucose homeostasis and perpetuating a vicious cycle of metabolic dysregulation.

Given the central role of lifestyle factors in the progression of MASLD and T2DM, dietary modifications and increased physical activity remain the cornerstones of disease management. Addressing modifiable risk factors, including obesity, poor diet, and physical inactivity, remains critical for preventing disease progression. Lifestyle modifications, including weight loss, dietary adjustments, and increased physical activity, have demonstrated significant benefits in both MASLD and T2DM. Current therapeutic approaches target IR, hepatic lipid accumulation, and inflammation, yet optimal pharmacological interventions remain under investigation.

Achieving at least 10% weight loss through lifestyle intervention is the most effective strategy for histological improvement of MASLD, including resolution of steatohepatitis and regression of fibrosis. For patients with obesity refractory to lifestyle measures, bariatric surgery has demonstrated sustained weight reduction and reversal of MASLD progression. Pharmacologic approaches are also advancing: pioglitazone has shown efficacy in improving steatohepatitis (249); incretin-based therapies such as liraglutide and semaglutide significantly improve steatosis, inflammation, and fibrosis (250, 251); and the dual GLP-1/GIP receptor agonist tirzepatide has demonstrated marked reduction in hepatic fat content and MASH resolution (203). Sodium-glucose cotransporter-2 (SGLT2) inhibitors are also emerging as promising agents that reduce liver fat and improve cardiometabolic outcomes. These developments illustrate how lifestyle interventions, metabolic surgery, and diabetes medications together form an expanding therapeutic toolkit for MASLD in the context of T2DM (252–255).

Despite advancements in understanding the pathophysiological mechanisms linking MASLD to T2DM, significant gaps persist, including the lack of standardized screening guidelines and the absence of targeted pharmacotherapies. Future research should focus on developing personalized treatment strategies based on genetic and molecular signatures. As the global burden of metabolic diseases rises, a multidisciplinary approach involving hepatologists, endocrinologists, and public health professionals will be essential to mitigate the impact of MASLD and T2DM on global health.

To mitigate the rising burden of MASLD and T2DM, a multidisciplinary and proactive approach is essential. Collaboration between hepatologists, endocrinologists, and public health experts can facilitate early identification, risk stratification, and intervention. Recognizing MASLD as a central component of metabolic disease is crucial for preventing its progression to T2DM and enhancing long-term patient outcomes and quality of life.
